# Temporal Effects of Cyclic Stretching on Distribution and Gene Expression of Integrin and Cytoskeleton by Ligament Fibroblasts In Vitro

**DOI:** 10.1080/03008200902846270

**Published:** 2009-07-27

**Authors:** Daiki Kaneko, Yoshihiro Sasazaki, Toshiyuki Kikuchi, Takeshi Ono, Kohichi Nemoto, Hideo Matsumoto, Yoshiaki Toyama

**Affiliations:** Department of Orthopaedic Surgery, National Defense Medical College, Saitama, Japan; Clinical Research Center, National Hospital Organization Murayama Medical Center, Tokyo, Japan; Department of Global Infectious Diseases and tropical Medicine, National Defense Medical College, Saitama, Japan; Department of Orthopaedic Surgery, National Defense Medical College, Saitama, Japan; Department of Orthopaedic Surgery, Keio University, Tokyo, Japan

**Keywords:** Cyclic Stretching, Integrin, Ligament Fibroblast, Cytoskeleton, Collagen

## Abstract

Cyclic stretching is pivotal to maintenance of the ligaments. However, it is still not clear when ligament fibroblasts switch on expression of genes related to the mechanotransduction pathway in response to cyclic stretching. This in vitro study investigated, using ligament fibroblasts, the time-dependent changes in distribution and gene expression of β1 integrin, the cytoskeleton, and collagens after the application of 6% cyclic stretching at a frequency of 0.1 Hz for 3 hr on silicon membranes. We carried out confocal laser scanning microscopy to demonstrate changes in distribution of these components as well as quantitative real-time RT-PCR to quantify levels of these gene expression both during application of cyclic stretching and at 0, 2, 6, 12, and 18 hr after the termination of stretching. Control (unstretched) cells were used at each time point. Within 1 hr of the application of stretching, the fibroblasts and their actin stress fibers became aligned in a direction perpendicular to the major axis of stretch, whereas control (unstretched) cells were randomly distributed. In response to cyclic stretching, upregulation of actin at the mRNA level was first observed within 1 hr after the onset of stretching, while upregulation of β1 integrin and type I and type III collagens was observed between 2 and 12 hr after the termination of stretching. These results indicate that the fibroblasts quickly modify their morphology in response to cyclic stretching, and subsequently they upregulate the expression of genes related to the mechanotransduction pathway mainly during the resting period after the termination of stretching.

## INTRODUCTION

The ligament is a highly organized tissue whose structure and metabolism are maintained by mechanical stimuli applied during daily activities. The highly oriented organization of collagens in the ligament is maintained by the fibroblasts joined along their longitudinal rows by longitudinally organized actin stress fibers linked end-to-end with adherent junctions [1, 2]. Thus, the mechanical stretching applied to the ligament in vivo is transferred throughout the ligaments and is thought to play some important roles in ligament morphogenesis and maintenance [2, 3]. At the cellular level, it has been suggested that mechanical stretching is transferred through the extracellular matrix (ECM) and into the cells across cell surface integrins and then through the cytoskeleton (CSK) with resultant modifications to cellular structure and metabolism [4–7].

Several studies have investigated the effects of cyclic stretching on the structure and metabolism of fibroblasts in vitro. When fibroblasts were seeded as a monolayer on deformable membranes and then subjected to cyclic stretching, the cells and their actin microfilaments became aligned in a direction almost perpendicular to the major axis of stretch [8–15]. There were two possible explanations for the mechanism of cell reorientation: re-redistribution of integrin and the CSK and their de novo expression from the mRNA level. Since it has been reported that morphological changes undergone by the fibroblasts in response to cyclic stretching can be observed immediately after the application of cyclic stretching, the morphological change seems to occur before upregulation of the genes encoding integrin and the CSK [8, 9–17]. However, the way in which the cells alter their orientation is still not fully understood.

Several studies have reported that in response to cyclic stretching, ligament fibroblasts increase the expression of genes encoding both cellular and extracellular protein components involved in mechanotransduction, including several subtypes of integrins, actin microfilaments, type I collagen, and type III collagen [18–24]. However, an in vivo study demonstrated that gene expression of type I collagen was upregulated within 1 hr from the onset of cyclic stretching and subsequently downregulated after 2 hr [24]. Another study also reported that in response to cyclic stretching, fibroblasts gradually downregulated expression of the gene encoding type I procollagen after application of cyclic stretching for 12 hr [21]. These observations suggest that application of prolonged cyclic stretching downregulates the expression of genes encoding ECM components.

Therefore, we hypothesized that a resting period after the application of cyclic stretching was required for the ligaments to promote their expression of genes encoding the CSK, integrin, and collagens. The hypothesis was tested by investigating the time-dependent effects of cyclic stretching on both the distribution of β1 integrins, actin, and vimentin and the expression of genes encoding such components as well as type I and type III collagens.

## MATERIALS AND METHODS

### Preparation of Ligament Fibroblasts

This study was approved by the Committee of the Center for Laboratory Animal Science, National Defense Medical College. Under diethyl ether anesthesia, the midsubstance of the anterior cruciate ligament was surgically dissected from Japanese white rabbits used for other studies approved by the National Defense Medical College, and chopped into small pieces under aseptic conditions. These pieces of ligament were treated with 0.25% actinase E (Kaken, Tokyo, Japan) for 1 hr and 0.025% collagenase type 1-A (Sigma-Aldrich, St. Louis, MO, USA) for 3 hr at 37°C. The resulting digest was centrifuged at 1500 rpm for 10 min and the cell pellet was suspended in serum-free Dulbecco's modified eagle medium (DMEM). The cells were washed with phosphate buffered saline (PBS) and resuspended in DMEM containing 10% fetal bovine serum (FBS) and 1% antibiotic/antimycotic solution. The cells were plated in petri dishes and maintained at 37°C in a humidified atmosphere of 5% CO_2_ in air. The medium was changed twice per week.

### Application of Cyclic Uniaxial Stretching

**FIG. 1:**
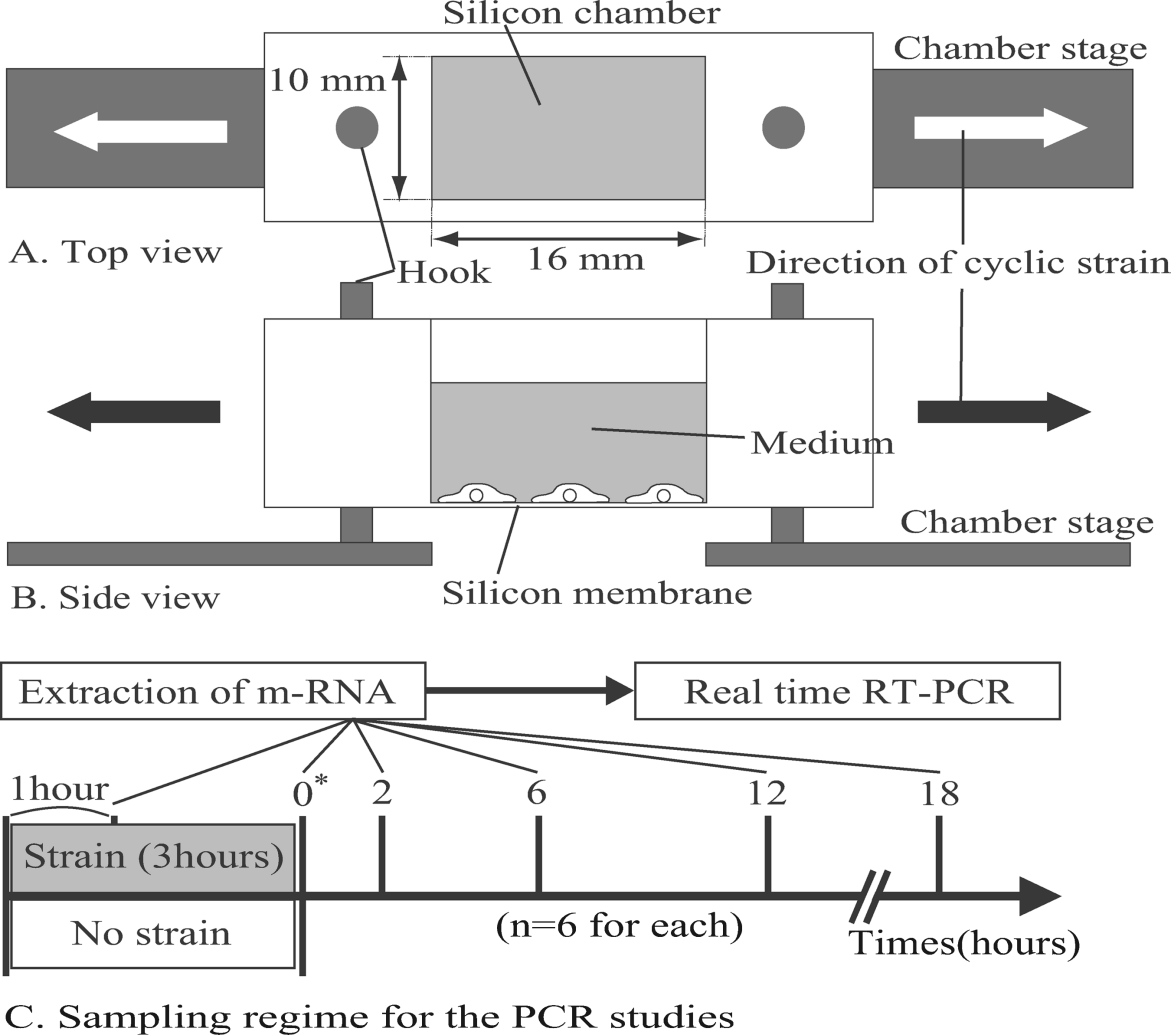
Top view (A) and side view (B) of a deformable silicon-well chamber containing a 16-mm-long × 10-mm-wide silicon membrane at its base. Ten wells are able to be simultaneously mounted on a multistation cell-stretching device. Arrows indicate the direction of applied stretching. (C) Sampling regime for the PCR studies. * Time 0 is defined as the point at which the stretching was terminated.

The cells were plated in deformable silicon-membrane chambers (Figure 1A, 1B) coated with type I collagen (Nitta gelatin, Osaka, Japan) at an initial density of 1 × 10^4^ cells/cm^2^ and allowed to attach for 24 hr before application of cyclic stretching. Since these chambers have hard silicon parts surrounding the thin silicon membrane, approximately pure uniaxial stretching could be applied on the membrane at 6% stretch without causing heterogeneous deformation which might result in membrane center narrowing and lateral wall thinning of the silicon well. Two groups of cells were prepared: in one, designated the stretched group, the cells within the chambers were mounted on a cell-stretch device (ST-140; STREX, Osaka, Japan) (Figure 1A, 1B) and 6% uniaxial cyclic stretching was applied at a frequency of 0.1 Hz for a 3-hr regimen in the incubator and then maintained at rest. In the other group, designated the control group (unstretched), the cells in the chambers were not subjected to stretching.

### Immunofluorescence Staining

The cells from both groups were fixed with 4% (w/v) paraformaldehyde and incubated with 1% bovine serum albumin (BSA). The extracellular domain of β1 integrins was labelled with an FITC-conjugated mouse monoclonal antibody to β1 integrin (Chemicon International, Temecula, CA, USA). Filamentous actin was labelled with Alexa-Fluor-633 conjugated to phalloidin (Invitrogen™, San Diego, CA, USA). Vimentin intermediate filaments were labelled with Cy3 conjugated to an antivimentin antibody (Sigma-Aldrich). Nuclei were counterstained with 120 μM DAPI (Invitrogen). Each silicon membrane was removed from the silicon chambers using a scalpel, mounted on a glass slide cell-side up, and covered with a cover slip using an antifade reagent (Vector Laboratories, Burlingame, CA, USA). The results of the antibody alone were used as negative control.

### Confocal Laser Scanning Microscopy

Confocal laser scanning microscopy was performed using a Zeiss-510 Meta (Carl Zeiss, Göttingen, Germany) to visualize the time-dependent effects of cyclic stretching on cell morphology and distribution of β1 integrins, actin, and vimentin. Two air lenses (10×/0.30 and 20×/0.80, Carl Zeiss) and two oil-immersion lenses (63×/1.40 and 100×/1.40, Carl Zeiss) were used for observations. A 405 nm blue diode laser, a 488 nm argon laser and helium-neon lasers emitting at 543 nm and 633 nm were set to excite DAPI (nucleus), FITC (β1 integrin), Cy3 (vimentin), and Alexa Fluor 633 (actin), respectively. The Z-series of fluorescence images of β1 integrins, actin, vimentin, and nuclei were acquired simultaneously using four emission-detectors and reconstructed three-dimensionally using software (LSM Image browser, Carl Zeiss).

### Quantitative Real-Time Reverse Transcription (RT)-Polymerase Chain Reaction (PCR)

Total RNA was extracted from both the control (unstretched) and the stretched group using an RNeasy plus mini kit (Qiagen, Valencia, CA, USA) following the manufacturer's protocol, both during stretching and at 0, 2, 6, 12, and 18 hr after the termination of stretching (Figure 1C). Time 0 in this study was defined as the time point of the termination of stretching. First, 25 Ng of total RNA per 20 μl of reaction volume were reverse transcribed into cDNA using a high capacity transcription kit (Applied Biosystems [ABI], Foster City, CA, USA]. Second, single-stranded cDNA products were then analyzed with real-time PCR using the TaqMan gene expression assays (ABI) and ABI Prism 7900HT sequence detector. cDNA samples (1 μl in a total reaction volume of 20 μl) were analyzed for the genes of interest using GAPDH as the housekeeping gene. The master-mix consisted of 8 μl of dH_2_0, 10 μl of TaqMan fast universal PCR master-mix (ABI), and 1 μl of PCR primers (Invitrogen). The level of expression of each target gene was normalized to GAPDH and then compared. Analysis of each sample was repeated 6 times for each gene of interest. RT-PCR was performed at 95°C for 2 min followed by 34 cycles of 30 sec of denaturation at 95°C, 30 sec annealing at the primer-specific temperature, and 1 min elongation at 72°C.

The PCR primers were β1 integrin (ITGB), β actin (ACTB), type I collagen alpha1 (COL1A1), type I collagen alpha2 (COL1A2), and type III collagen alpha1 (COL3A1) (Table 1). The primer sequences were obtained using Blat (http://genome.ucsc.edu/cgi-bin/hgBlat) in UCSC (http://genome.ucsc.edu) from the target genes of human and mouse homologues. Thereafter the primer sequences and probes were designed using Primer3 (http://primer3.sourceforge.net/). For comparison of expression of each gene at each time point between the stretched and control groups, the data were analyzed using Student's *t*-test. Statistical significance was determined at the *p* 0.05 level for all tests.

**TABLE 1:** Primers specific for β actin (ACTB), β1 integrin (ITGB), type-I collagen alpha1 (COL1A1), type-I collagen alpha2 (COL1A2), type-III collagen alpha1 (COL3A1), and GAPDH control

	Sense	Antisense
ACTB	5′-GGC CGT CTT CCC CTC CAT CG-3′	5′-CTA GTT GGT CAC GAT GCC GTG C-3′
ITGB	5′-CAG GCA GGC CCC AAT TGT GG-3′	5′-CCT TTG CTA CGG TTG GTG ACA TT-3′
COL1A1	5′-CAT CAA GGT CTT CTG CGA CA-3′	5′-CTT GGG GTT CTT GCT GAT GT-3′
COL1A2	5′-CAA TCA CGC CTC TCA GAA CA-3′	5′-TCG GCA ACA AGT TCA ACA TC-3′
COL3A1	5′-GCT CCT GGA CAG AAT GGT GAG-3′	5′-CGC CTT TGA CAC CTT GAG GA-3′
GAPDH	5′-GTG AAG GTC GGA GTG AAC G-3′	5′-CAA CAT CCA CTT TGC CAG AGT TAA-3′

## RESULTS

### Distribution of β1 Integrins and the Cytoskeleton

**FIG. 2:**
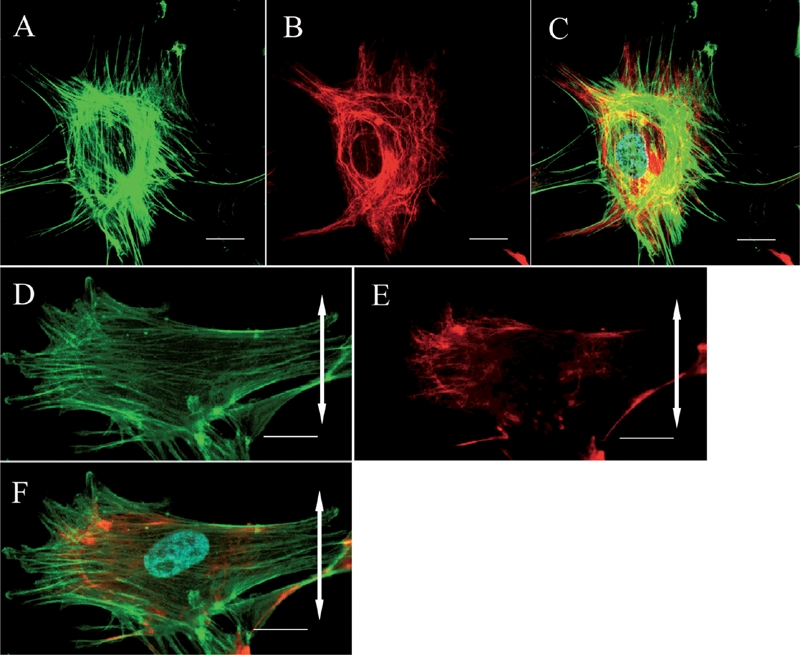
Confocal laser scanning microscopy. The cytoskeletal organization of a ligament fibroblast in the control group (A, B, C) and the stretched group (D, E, F) (Bar: 20 μm). The double-headed arrows represent the direction of applied stretching. In the control group, the actin stress fibers (A: green) and vimentin meshwork (B: red) are distributed randomly. (C) Merged image (nucleus: cyan). After stretching, the actin stress fibers (D: green) and vimentin meshwork (E: red) are oriented parallel to the longitudinal axes of the cells. (F) Merged image.

**FIG. 3:**
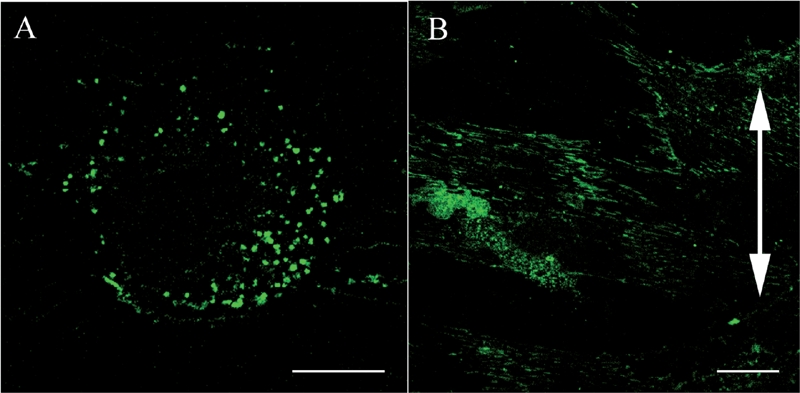
Confocal laser scanning microscopy. Effects of cyclic stretching on the distribution of β1 integrins within ligament fibroblasts (Bar: 20 μm). (A) In the control group, the β1 integrins (green) are randomly distributed within the plasma membrane attached to the silicon substrate and have a dot-like appearance. (B) In the stretched group, the β1 integrins are strongly distributed on the polar sides of the stretched cells and have a linear shape. The double-headed arrow indicates the direction of applied stretching.

In the control group, fibroblasts were randomly aligned and exhibited a variety of shapes. Actin stress fibers were distributed randomly and vimentin formed a random meshwork which was distributed between the plasma membrane and the nuclear membrane (Figure 2A–2C). In the stretched group, fibroblasts became aligned away from the direction of applied stretching, while parallel arrays of actin stress fibers and a vimentin meshwork within the individual fibroblasts also became aligned in this direction (Figure 2D–2F). β1 integrins in fibroblasts from the control group were distributed randomly within the plasma membrane attached to the silicon membranes and showed a dot-like appearance (Figure 3A).

**FIG. 4:**
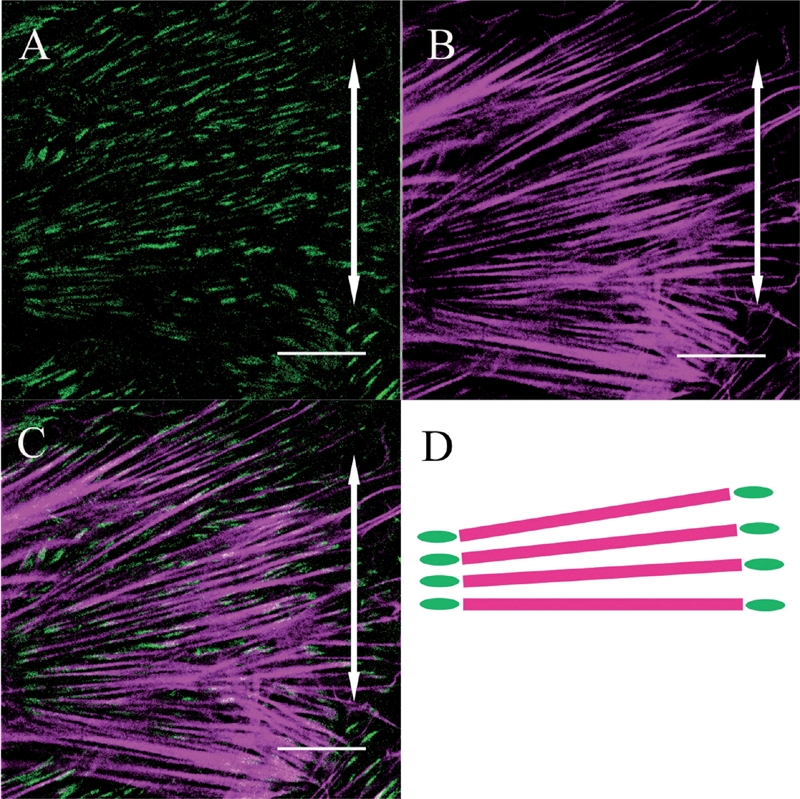
Both β1 integrin (A) and actin stress fibers (B) within a fibroblast are aligned perpendicular to the direction of applied stretching (double-headed arrows). The merged image (C) and its schematic drawing (D) show that β1 integrin is distributed at both ends of the actin stress fibers (Bar: 20 μm).

Meanwhile, in fibroblasts from the stretched group, β1 integrins were heavily distributed on the polar sides of the fibroblasts and were linear in shape aligned parallel to the cells' long axes (Figure 3B). A merged image of β1 integrins and actin in the stretched group fibroblasts demonstrated that β1 integrins were distributed at both ends of the actin stress fibers which were aligned away from the direction of applied stretching (Figure 4A–4D).

### Gene Expression

**FIG. 5:**
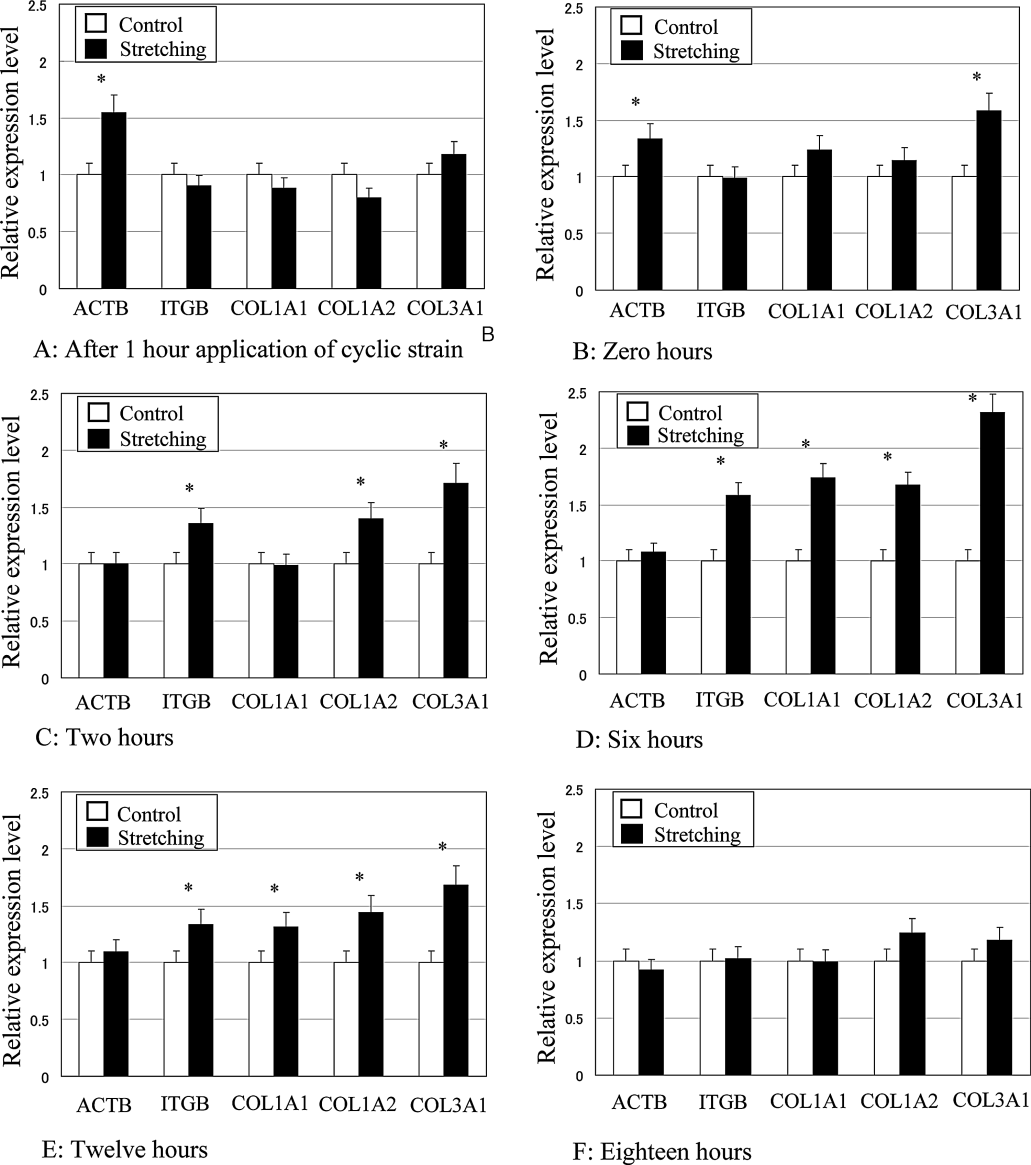
Time-dependent effects of cyclic tensile stretching on the gene expressions of β actin (ACTB), β1 integrin (ITGB), type-I collagen alpha1 (COL1A1), type I collagen alpha2 (COL1A2), and type III collagen alpha1 (COL3A1) in ligament fibroblasts. The relative expression level represents the target gene expression relative to GAPDH in the stretched group normalized to the control group. * *p* < 0.05.

The gene expression of β1 integrin, β actin, and type I and type III collagens relative to GAPDH became upregulated compared to control in response to cyclic tensile stretching in a time-dependent manner. After 1 hr from the application of cyclic stretching (Figure 5A), expression of β actin in the stretched fibroblasts was 55% higher than that in the unstretched control group, and this upregulation was observed until the termination of cyclic stretching. After cyclic stretching had been applied for 3 hr (Figure 5B), the expression of type III collagen in the stretched group was 58% higher than that in the unstretched control. At 2 hr after the termination of cyclic stretching (Figure 5C), expression of β1 integrin, type I collagen alpha2, and type III collagen in the stretched group were 35%, 40%, and 71% higher than those of control, respectively. At 6 hr after the termination of stretching (Figure 5D), ITGB, COL1A1, COL1A2, and type III collagen in the stretched group were 74%, 58%, 67%, and 131% higher than that of control, respectively. Up to 12 hr after the termination of cyclic stretching (Figure 5E), β1 integrin, type I, and type III collagens were still upregulated in response to cyclic stretching. However, at 18 hr after the termination of stretching, no upregulation of gene expression was observed (Figure 5F).

## DISCUSSION

This study supports the idea that ligament cells change their morphology and orientation before upregulation of the genes encoding both cellular and extracellular components involved in mechanotransduction, i.e., β1 integrins, β-actin, type I collagen, and type III collagen. Previous studies have shown that several subtypes of integrin are upregulated in response to cyclic tensile stretching and several alternative mechanisms have been proposed: an increase in integrins at the mRNA level or an increase in integrin recruitment and/or recycling at the cell surface [18, 19, 21, 22, 25–28]. These studies also suggested that some families of genes appeared to control the recycling of integrin and actin stress fibers to modify the lamellipodium and filopodium—rather than controlling these processes by upregulation of target genes at the mRNA level. Some studies reported that Rho family and Rab family proteins played important roles in actin repolymerization and recycling of integrins, respectively [26–28]. Since the present study demonstrated that morphological changes occurred within 1 hr after the onset of cyclic stretching and before upregulation of β1-integrin at the mRNA level, the early re-distribution of β1 integrin might be due to its recycling. Several studies have described that in response to the application of cyclic stretching, the cells become oriented nearly perpendicular to the stretching direction and that the cells reorganize their shape to minimize excessive stretching or deformation in the direction of the major axis of stretch [8–15]. However, Wang et al. [15–17] demonstrated that cells which were aligned in the direction of silicon microgrooves did not change their alignment even after the application of cyclic stretching.

These previous observations suggested that both contact guidance and the direction of applied stretching determine cellular orientation and shape. In this study, we demonstrated that this cellular realignment was accompanied by coordinate changes in the distribution of β1 integrins and the β actin and vimentin meshwork within each cell. The cells seemed to perceive force direction quickly and subsequently initiate the rearrangement of their β1 integrins and actin coordinately to the appropriate regions of the individual cells, subsequently upregulating expression of the genes encoding β1 integrins and collagens.

This study also supports the hypothesis that a resting period after the termination of cyclic stretching is required for the ligament fibroblasts to upregulate the genes encoding both cellular and extracellular components involved in mechanotransduction. Previous studies demonstrated that type 1 collagen gene expression was gradually down regulated during continuous cyclic stretching both in vivo and in vitro [21, 24]. These facts suggested that the fibroblasts increase synthesis of collagen during the resting period after the termination of cyclic stretching, while they suppress it during the application of excessive continuous stretching. We observed that the upregulation of β1 integrins and type I and type III collagens occurred during the resting period after the termination of cyclic stretching and this lasted for at least 10 hr. Our results suggest that the resting period after the termination of cyclic stretching might be the critical period for ligament fibroblasts to increase expression of genes involved in mechanotransduction and the synthesis of these proteins.
